# Putative morphology of *Neoehrlichia mikurensis* in salivary glands of *Ixodes ricinus*

**DOI:** 10.1038/s41598-020-72953-0

**Published:** 2020-09-29

**Authors:** Jaroslav Ondruš, Pavel Kulich, Oldřich Sychra, Pavel Široký

**Affiliations:** 1 Department of Biology and Wildlife Diseases, Faculty of Veterinary Hygiene and Ecology, University of Veterinary and Pharmaceutical Sciences Brno, Palackého tř. 1946/1, 612 42 Brno, Czech Republic; 2 CEITEC, Central European Institute of Technology, University of Veterinary and Pharmaceutical Sciences Brno, Palackého tř. 1946/1, 612 42 Brno, Czech Republic; 3 Department of Pharmacology and Toxicology, Veterinary Research Institute, Hudcova 296/70, 621 00 Brno, Czech Republic

**Keywords:** Infectious diseases, Transmission electron microscopy, Bacteria, Pathogens

## Abstract

*Neoehrlichia mikurensis* is an emerging tick-borne intracellular pathogen causing neoehrlichiosis. Its putative morphology was described in mammalian, but not in tick cells. In this study, we aim to show the presumptive morphology of *N. mikurensis* in salivary glands of engorged females of *Ixodes ricinus*. To accomplish this, we collected *I. ricinus* ticks in a locality with a high *N. mikurensis* prevalence, allowed them to feed in the artificial in vitro feeding system, dissected salivary glands and screened them by PCR for *N. mikurensis* and related bacteria. Ultrathin sections of salivary glands positive for *N. mikurensis* but negative for other pathogens were prepared and examined by transmission electron microscopy. We observed two individual organisms strongly resembling *N. mikurensis* in mammalian cells as described previously. Both bacteria were of ovoid shape between 0.5–0.8 μm surrounded by the inner cytoplasmic and the rippled outer membrane separated by an irregular electron-lucent periplasmic space. Detection of *N. mikurensis* in salivary glands of *I. ricinus* suggests that this bacterium uses the “salivary pathway of transmission” to infect mammals.

## Introduction

*Neoehrlichia mikurensis* is a gram-negative intracellular bacterium in the family Anaplasmataceae, widespread among the ticks in Europe and Asia^1,2^. In Europe, *Ixodes ricinus* (Linnaeus, 1758) is the primary vector^1^ and rodents seem to be the main reservoir hosts^3^. Even though *N. mikurensis* was discovered in 1999^4^, limited information is available regarding its life cycle, mechanisms of cell infiltration and morphology.

In infected humans, *N. mikurensis* can induce “neoehrlichiosis”. Severity and symptoms of this novel disease vary greatly, from a mild illness^5^ to serious infections^6,7^ characterized by fever and vascular events endangering mainly individuals with immune-compromising conditions^2^. Asymptomatic courses of infection were also reported^8^. Nonetheless, most cases are probably misdiagnosed or not diagnosed at all since *N. mikurensis* cannot be currently detected by conventional approaches, but only by PCR^2^. Recently, Wass et al. (2019) succeeded in cultivation of this bacterium for the first time, propagating *N. mikurensis* in embryonal *Ixodes* spp. cell lines IRE/CTVM20 and ISE6^9^. Therefore, it is no longer necessary to use the prefix “*Candidatus*”.

Regarding the morphology and ultrastructure of *N. mikurensis*, two papers were published^10,11^. However, both of them showed suspected *N. mikurensis* in mammalian cells. Some bacterial species related to *N. mikurensis*, e. g. *Ehrlichia chaffeensis,* are known to have different morphology in mammalian and tick cells^12^. Therefore, our goal in this study was to show the morphology *N. mikurensis* in salivary glands of the tick *I. ricinus*.

## Material and methods

### Tick collection

Questing adult *I. ricinus* were collected in April 2018 by flagging^13^ from the vegetation near Kvítkův Dvůr, Český Krumlov, Czech Republic (48.8093514 N, 14.2936328E), locality identified as a *N. mikurensis* hotspot in our previous large-scale epidemiological study, where 13,325 questing *I. ricinus* ticks from 140 areas throughout the Czech Republic were analyzed for the presence of *N. mikurensis*^14^. Respecting the sex, ticks were stored in 50 ml Falcon Tubes supplied with 4–6 grass blades and transported to the laboratory.

### In vitro feeding

Sampled ticks were allowed to engorge in a modified artificial in vitro feeding system^15^. In contrast to the original protocol, we did not use chemical stimuli and hair. The collected ticks were equally distributed into 12 feeding wells, so that each contained 9–10 females and the same number of males. The bovine blood used was collected at a local abattoir and immediately supplied with heparin (15 U/ml), Nystatin (100 U/ml), Gentamicin (5 µg/ml), Adenosine 5′-triphosphate (1 mM) and glucose (4 g/ml) (all chemicals purchased at Sigma-Aldrich, Darmstadt, Germany). Blood exchanges were performed every 11–13 h throughout the whole course of feeding, which took place for 9 days. Ticks were fed in a water bath WNB 29 (Memmert GmbH, Schwabach, Germany) set to 36 °C.

### Acquisition of salivary glands

Engorged females were embedded in a wax-filled Petri dish. For each tick, the dorsal part of the cuticle covering the idiosoma was removed using Self-Opening Mini-Micro Tweezers (Excelta, Buellton, USA). Opened ticks were rinsed thoroughly with sterile Phosphate-buffered saline (PBS). Next, midgut, Malpighian tubules and ovaries were individually removed, washed and stored for an unrelated study. Remnants of the blood meal were continuously washed away by PBS during the whole course of dissection. One salivary gland of each tick was subjected to immediate DNA extraction and subsequent PCR, while the second gland was put into 3% glutaraldehyde and later processed into ultrathin sections.

### DNA extraction and PCR

DNA was extracted using a NucleoSpin Tissue kit (Macherey–Nagel, Düren, Germany) following the manufacturer's protocol. After the extraction, concentration and purity were checked by an Implen NanoPhotometer P330 (Implen, Munich, Germany) and stored at − 20 °C. Conventional PCR detections of *I. ricinus*, *N. mikurensis* and related bacteria were carried out using PPP Master Mix according to the manufacturer's instructions (Top-Bio s.r.o., Vestec, Czech Republic). Primer names, sequences, annealing temperatures, amplicon lengths and sequences used for the primer design are listed in Table 1. Primers were designed with the Geneious 11.1.4 software (https://www.geneious.com). The PCR products were separated electrophoretically in 1.5% agarose gels stained with Midori Green (Elisabeth Pharmacon, Brno, Czech Republic) and visualized under UV light. Specificity of the PCR reaction was confirmed by Sanger sequencing using the same primer set as for the detections^14^.

**Table 1:** Tested organisms in salivary glands and used primers.

Organism	Numbers of positive to examined glands	Primer names	Reference	Sequence	Annealing temperature	Amplicon length	Designed according to sequences
*Ixodes ricinus*	73/73	Ire_16S_PCR_F	This study	5′-CTGTGGTATTTTGACTATACGAAGG-3′	61 °C	310 bp	KY319188, KM211788, KM211785, GU074591, IXOMTRGDG
		Ire_16S_PCR_R		5′-TCCAAAATTATTACGCTGTTATCCC-3′			
*Neoehrlichia mikurensis*	7/73	CNM_groEL_PCR_F	^14^	5′-AACTACAACATGTTCTATTTTAACAGC-3′	52.4 °C	654 bp	JQ359062, KX980040, FJ966359, KF803997, EU810406, AB074461, AB084583, HM045824
		CNM_groEL_PCR_R		5′-TCGTCATTAATAACGTATTTTGCACC-3′			
*Rickettsia* spp.	1/7	Rick_gltA_PCR_F	This study	5′-AAAGGAATCTTGCGGCATCG-3′	57.2 °C	581 bp	KX159435, AF201330, KU961540, KU961539, KP866150, AF178035, DQ402516
		Rick_gltA_PCR_R		5′-GCAATACCCGTGCTAATACAAGC-3′			
*Ehrlichia* spp.	0/7	Ehr_groEL_PCR_F	This study	5′-GCTGGACCTAAAGGGCTTACTG-3′	57.2 °C	123 bp	AF195273, JN391408, KX987388, GU358686, AB032711
		Ehr_groEL_PCR_R		5′-AGCAATAGCAAGAGCCAATGGA-3′			

### Transmission electron microscopy

The salivary glands were left in 3% glutaraldehyde for 5 days. Next, glands were washed three times for 10 min in 0.1 M Millonig's Phosphate buffer and contrasted in 2% OsO_4_ for 60 min. After contrasting, the glands were dehydrated through a graded series of acetone at room temperature, unless specified otherwise: 30% 20 min, 50% 20 min, 70% overnight at 4 °C, 90% 30 min, and twice with 100% for 30 min. Next, samples were embedded in Epon/Durcupan (E/D) mixture according to the manufacturer's recommendations (all used chemicals were purchased from SERVA Electrophoresis GmbH, Heidelberg, Germany). This mixture was further mixed with 100% acetone 1:1 and 3:1 and samples were incubated in both solutions for 45 min. Next, samples were incubated in pure E/D solution 3 × 60 min. After this step, samples were put into gelatin capsules and left to polymerize at 60 °C for 4 days. Blocks were trimmed on Leica EM TRIM2 and sectioned (60 nm) on the ultramicrotome Leica UC 7 (both machines purchased from Leica Microsystems, Vienna, Austria). Ultrathin sections were observed in EM Philips 208 S Morgagni (FEI, Brno, Czech Republic) at an accelerating voltage of 80 kV.

### Ethics approval

This study did not include any experiments in humans, vertebrates neither higher invertebrate subjects. Therefore, all experiments were conducted in compliance with relevant European Union guidelines (86/609/EEC) and with the Czech national legislation on the use of animals and protection of animals against cruelty (Animal Welfare Act No. 246/1992 Coll.). Thus, it did not require any approval of institutional animal ethical committee. All methods and experimental protocols in the study were carried out in accordance with institutional guidelines.

## Results and discussion

In total, 73/118 *I. ricinus* females engorged in the in vitro feeding system and were dissected. DNA of *N. mikurensis* was detected in 7/73 salivary glands (Table 1). All amplified sequences were identical to that found on the locality of flagging (Český Krumlov) previously^14^, which is available in GenBank under the accession code MN151364. One of the positive glands (1/7) was found to be co-infected with *Rickettsia* spp. Sequencing revealed 99.82% identity with the *Rickettsia helvetica* on a 558-bp-long fragment of the *gltA* gene, which can be viewed under the accession code KU310588.1. Therefore, corresponding embedded gland was excluded from the study. None of the *N. mikurensis* positive salivary glands tested positive for *Ehrlichia* spp. (Table 1) and none was further examined for the presence of other bacteria from the order Rickettsiales.

Detection of *N. mikurensis* in salivary glands suggests that this bacterium uses the “saliva transmission pathway”, which is generally acknowledged as a transmission route of most tick-borne pathogens^16^ including other members of the family Anaplasmataceae, e. g. *Anaplasma marginale*^17^. However, some other tick-borne pathogens are also transmitted by an alternative route, when the ingested blood is mixed with the bacteria present in the midgut acquired in the previous life stage and regurgitated to the host during feeding^18^. To the best of our knowledge, no research concerning the mechanism of infection and the pathogen distribution in tick organs has been conducted. Therefore, confirmation of the salivary transmission pathway of *N. mikurensis* and its distribution in individual tick organs by additional research, utilizing, for example, microscopic tracking and in situ hybridization as was shown previously^19,20^ is necessary.

Also, it should be noted that detection of *N. mikurensis* in salivary glands of *I. ricinus* fed on the blood treated with Gentamicin does not automatically prove its resistance to this antibiotic. It may be that *N. mikurensis* bacteria were not alive, but retained their morphology and genome fragments at the time of dissection. However, there are several reports of human patients with neoehrlichiosis that showed no response to Gentamicin^21,22^, suggesting that *N. mikurensis* might not be affected by this antibiotic. Thus, further research is needed to answer the question of a possible resistance of *N. mikurensis* to Gentamicin.

**Figure 1:**
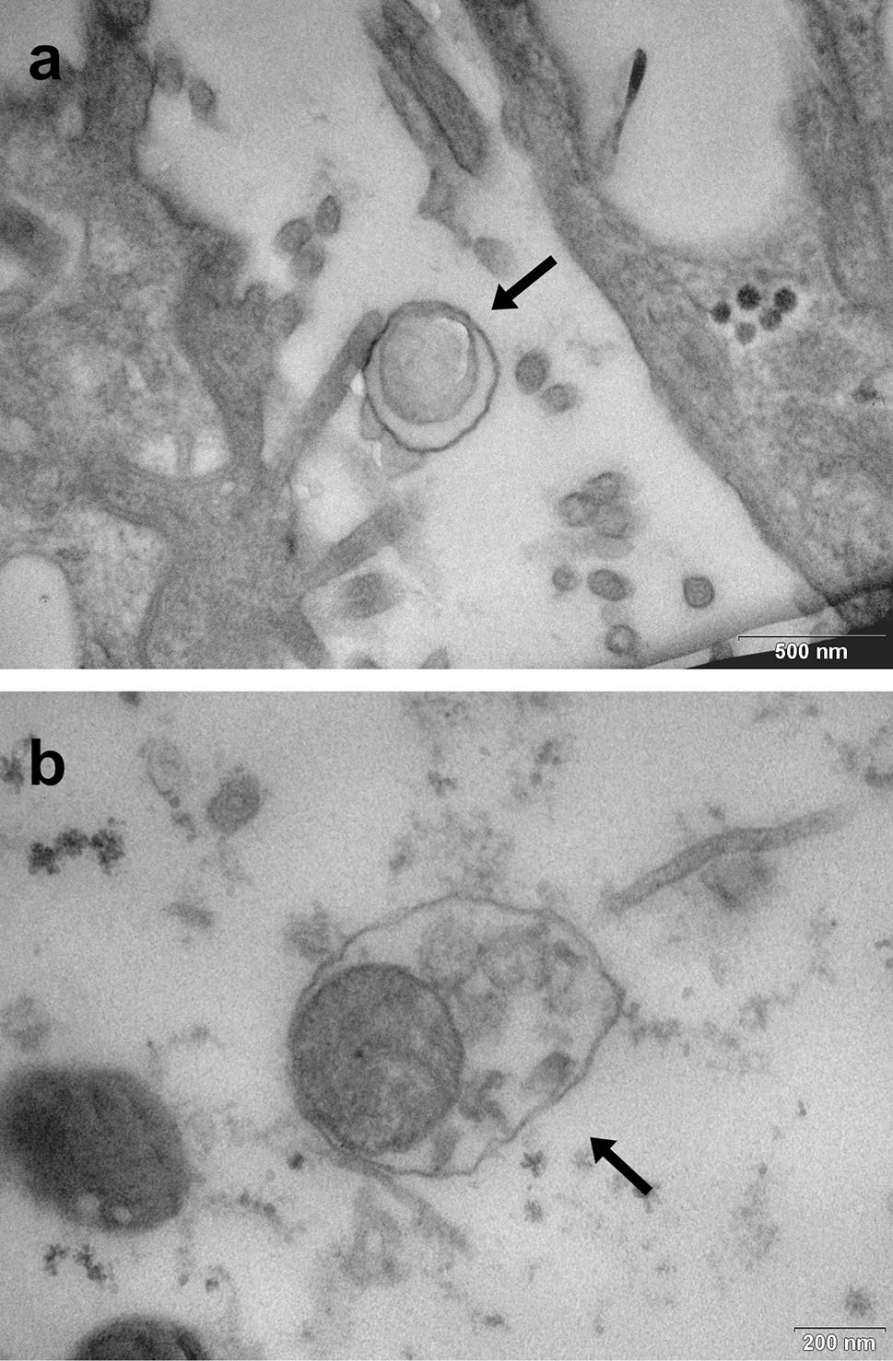
Transmission electron micrographs of suspected *Neoehrlichia mikurensis* in salivary glands of naturally infected engorged female *Ixodes ricinus*. Both individual organisms are surrounded by the rippled outer and the inner cytoplasmic membrane with electron-lucent periplasmic space. Scale bar: 500 nm (**a**), 200 nm (**b**).

We spotted two suspected *N. mikurensis* organisms (Fig. 1) in one of the infected salivary glands. Both of them contained a Gram-negative type cell wall consisting of the inner cytoplasmic and the rippled outer membrane separated by an irregular periplasmic space, which is characteristic of the family Anaplasmataceae^23^. Both bacteria were of an ovoid shape with a diameter of 0.5–0.8 μm. These attributes correspond to the suspected *N. mikurensis* described previously in endothelial cells of Wister rats^10^ and human granulocytes^11^. Interestingly, the latter paper reported the finding of individual bacteria, which were also observed in our study. In contrast, Wass et al. (2019) described aggregates of *N. mikurensis* tightly surrounding the nucleus in ISE6 cells^9^, the embryo-derived *I. scapularis* cell lines^24^. Presumably, these bacteria were packed in intravacuolar microcolonies, formations of numerous organisms typical of related intracellular bacteria, e. g. *Neoehrlichia lotoris*^25,26^, *Candidatus* Ehrlichia khabarensis^27^ and *Anaplasma phagocytophilum*^28^. On the contrary to our and the two previously published papers^10,11^ depicting putative *N. mikurensis*, Wass et al*.* (2019) used a labelled specific DNA probe^9^. Hence, their findings of bacterial colonies supported by a direct hybridization-based evidence can be considered reliable. However, our findings are not necessarily in opposition, as Wass et al. (2019) mostly screened heavily infected tick cell lines during the 9th week of culture^9^, while we worked with naturally infected salivary glands which were acquired within 5 days post detachment. Therefore, it may be that the numbers of the present bacteria were low at this point and morulae were not spotted or developed at the time of dissection.

The finding of only two putative *N. mikurensis* cells may be explained by different sensitivities of used methods of detection. In order to establish the level of detection of the PCR protocol, we designed gBlock covering the target area of the *groEL* gene plus 50 nucleotides on both ends (see Supplementary Data 1 online). We found that the detection limit of the used PCR protocol is 1–10 copies of the *groEL* gene. Therefore, it seems that the numbers of *N. mikurensis* bacteria were sufficient for the PCR detection, while it was unlikely to spot the bacteria by transmission electron microscopy, which has a detection limit of 10^5^–10^6^ particles/mL (viral, smaller, but comparable particles)^29^.

In conclusion, our findings should be treated carefully, for we show 2 individual suspected *N. mikurensis* bacteria in salivary gland of infected engorged *I. ricinus* female which tested positive by PCR for this bacterium and negative for *Rickettsia* spp. and *Ehrlichia* spp. However, we do not have a direct evidence that the shown structures are truly *N. mikurensis*. Therefore, additional research is needed to confirm our observations. It would be beneficial if the morphology and the ultrastructure of *N. mikurensis* would be examined on infected tick cell lines. Still, we believe that our findings contribute to the body of knowledge of *N. mikurensis* in ticks.

## Supplementary information

Supplementary file1

## Data Availability

Data are available on request.
