# Supplementary material for: Putative morphology of Neoehrlichia mikurensis in salivary glands of Ixodes ricinus
Source: Sci Rep. 2020 Sep 29;10:15987. doi: 10.1038/s41598-020-72953-0 (PMC7525475; doi:10.1038/s41598-020-72953-0)
Supplement: Supplementary file 1 — Supplementary file1 [file 41598_2020_72953_MOESM1_ESM.pdf]

<u>Putative morphology of *Neoehrlichia mikurensis* in salivary glands of *Ixodes ricinus*</u>

Authors

Jaroslav Ondruš*, Pavel Kulich, Oldřich Sychra, Pavel Široký

Supplementary Data 1

This gBlock sequence (754 bp) covers the target area of the *groEL* gene of *Neoehrlichia mikurensis* (654 bp) plus 50 nucleotides on both ends. Primer annealing sites are highlighted in bold. Two nucleotide substitutions on positions 100 and 101 were introduced to be able to distinguish between the gBlock and a possible contamination with bacterial DNA. gBlock was purchased at KRD, Praha, Czech Republic.

5’AATATAATAGCTCAAAGTGCTTCACAGTGTAATGACAAAGTTGGTGATGG**AACTACAACATGTTCTATTTTA**
ACAGCTAAAGTAATCGAGGAGGTATCTttAGCTAAAGCTGCTGGAGCAGATATTATTAGTATCAAAAATGGTAT
CTTAAAAGCAAAGGAATTAGTATTAGAATCTTTACTTTCTATGAAACGTGATGTTTCTTCAGAAGATGAAATAG
CACAGGTTGCAACAATTTCTGCAAATGGAGATAAAAACATAGGTAGCAAAATTGCACAATGCGTAAAAGAAGT
TGGTAAAGATGGTGTTATTACAGTTGAAGAAAGTAAGGGATTTAAGGAGTTAGAAGTTGAAAAAACTGATGG
TATGCAATTTGATAGAGGTTATTTATCGCCATATTTTGTAACTAATGCAGAAAAAATGTTAATTGAATTTGAAAA
TCCATATATTTTATTAACAGAAAAAAAATTAAATATCATACAGCCAATATTACCTATCCTTGAAAATATAGCAAG
ATCAGGTAGACCTCTACTAATTATTGCTGAAGATGTAGAAGGTGAAGCATTTAGTACTTTAGTGCTAAATAAGT
TACGTGGTGGATTGCATGTTGCTGCTGTTAAGGCACCAGGATTTGGTGATAGAAGAAAAGATATGCTTGGTGA
TATTGCAATATTAACT**GGTGCAAAATACGTTATTAATGACGA**ATTAGCAGTTAAAATGGAAGATCTAACATTA
GATGATCTAGGTACTGCAA’3
